# NASAFYTOL^®^ supplementation in adults hospitalized with COVID-19 infection: results from an exploratory open-label randomized controlled trial

**DOI:** 10.3389/fnut.2023.1137407

**Published:** 2023-06-22

**Authors:** Jean Gérain, Melanie Uebelhoer, Bérénice Costes, Julie Herman, Sandra Pietri, Anne-Françoise Donneau, Justine Monseur, Yves Henrotin

**Affiliations:** ^1^Department of Internal Medicine, CHIREC Hospital Group, Brussels, Belgium; ^2^Artialis SA, Avenue Hippocrate 5, Liège, Belgium; ^3^Biostatitics Unit, Département des Sciences de la Santé Publique, Université de Liège, Liège, Belgium

**Keywords:** COVID-19, SARS–CoV-2, coronavirus, food supplement, curcumine, quercetine, vitamin D, clinical trial

## Abstract

**Objectives:**

The effect and safety of Nasafytol^®^, a food supplement combining curcumin, quercetin, and Vitamin D, on hospitalized COVID-19-positive patients as support to standard of care were to be assessed.

**Methods:**

This exploratory, open-label, randomized, controlled trial was carried out among hospitalized adults with COVID-19 infection. Participants were randomly assigned to receive Nasafytol^®^ or Fultium^®^ control. The improvement of the clinical condition and occurrence of (serious) adverse events were evaluated. The study was registered on clincaltrials.gov with the identifier NCT04844658.

**Results:**

Twenty-five patients received Nasafytol^®^, and 24 received Fultium^®^. Demographic characteristics were well balanced between the groups. On day 14 (or at hospital leave if < 14 days), no difference was observed between groups regarding their clinical condition, fever, or the need of oxygen therapy. At day 7, however, 19 participants had been discharged from the hospital in the Nasafytol^®^ arm compared to 10 participants in the Fultium^®^ arm. No participants were transferred to the ICU or died in the Nasafytol^®^ arm, vs. 4 transfers and 1 death in the Fultium^®^ arm. The clinical condition of participants in the Nasafytol^®^ arm had improved, as evidenced by a decrease in the COVID-19 WHO score. Interestingly, five SAEs occurred with Fultium^®^, while no SAE was observed with Nasafytol^®^.

**Conclusion:**

Supplementation with Nasafytol^®^, in addition to standard-of-care treatment, led to a faster discharge from the hospital, improved clinical conditions of participants, and a reduced risk of serious outcomes, including transfer to the intensive care unit or death, in patients hospitalized with COVID-19.

## 1. Introduction

Since its origin in China in 2019, the global pandemic of the novel coronavirus disease 2019 (COVID-19) caused by severe acute respiratory syndrome coronavirus 2 (SARS-CoV-2) has since spread worldwide and caused more than 6.2 million deaths, according to the COVID-19 dashboard 2021 (Available at: https://coronavirus.jhu.edu/map.html, Accessed June 01, 2022). A wide spectrum of symptoms can be observed, ranging from asymptomatic to critical symptoms including respiratory distress and death. While in the majority of the cases only mild symptoms are observed, 10–15% of patients show a moderate to severe course of the disease and require hospitalization and oxygen support (1).

Vaccination has since become the most effective means to prevent a serious course of the disease, with several different forms of SARS-CoV-2 vaccines available around the world. Inequities regarding vaccine availability between different countries and populations remain however an ongoing challenge (2). In addition, despite an ever-growing percentage of the population being fully vaccinated, hospitalizations due to severe COVID-19 symptoms remain high as new SARS-CoV-2 variants keep emerging. Identifying effective and widely available pharmacological treatments against COVID-19 is therefore of utmost importance. More than 2000 active clinical treatment trials are underway as of June 1, 2022 (clinicaltrial.gov Accessed June 01, 2022). The only drug approved by the Food and Drug Administration (FDA) for the treatment of COVID-19 so far is remdesivir. While no effect on mortality was observed for remdesivir in two independent clinical trials (3, 4), the time to clinical recovery in hospitalized patients was reduced in another large, placebo-controlled trial (5). Other antiviral drugs, such as ritonavir-boosted nirmatrelvir or molnupiravir, have received Emergency Use Authorizations from the FDA (NIH COVID-19 treatment guidelines), and anti-SARS-CoV-2 antibody products such as the interleukin-6 (IL-6) receptor inhibitor tocilizumab and immunomodulators, including corticosteroids, are also being evaluated for their efficacy in treating COVID-19 (6–8). The downsides of these treatment options are however the often-important side effects and possible drug–drug interactions, mainly for nirmatrelvir, with concomitant medications.

In addition to antiviral and immune-based therapies, supportive treatments like food supplements are of great interest for the prevention and/or treatment of COVID-19 because of their good safety profiles and their beneficial effect on the immune system (9). Several *in vitro* studies have demonstrated the potential role of Vitamin D in antiviral innate immunity, and a possible role of Vitamin D to decrease the risk of a COVID-19 infection and mortality has been described (10). However, in a recent clinical trial, a single high dose of Vitamin D was not able to significantly reduce the length of hospitalization in patients with COVID-19 (11).

Quercetin (3,3′,4′,5,7-pentahydroxyflavone) and curcumin [(1E,6E)-1,7-bis(4-hydroxy-3-methoxyphenyl)-1,6-heptadiene-3,5-dione] are flavonoids with antiviral, immunomodulatory, antibacterial, and antioxidant properties, having demonstrated their effectiveness on several strains of influenza A, hepatitis B and C, Ebola and the coronavirus *in vitro* (12–14). In mice, curcumin and quercetin have beneficial effects on pneumonia of viral origin and lead to an increased survival rate and reduced severity of pneumonia (15–17). Moreover, a recent review suggests that curcumin could be a potential treatment option for patients with coronavirus disease due to its antiviral, antipyretic, antiemetic, antioxidant and anti-inflammatory properties (18). Interestingly, curcumin showed beneficial immunomodulatory effects in a randomized, placebo-controlled study of patients with allergic rhinitis (19). Both curcumin and quercetin have great safety profiles. Supplementation with 4–8 g/day of curcumin or up to 1 g/day of quercetin for 3 months did not lead to any adverse effects (20, 21).

Most recently, a beneficial effect of a combination of curcumin, quercetin and vitamin D3 as an adjuvant therapy has been reported for mild to moderate early-stage COVID-19 symptoms in ambulatory patients (22). In line with these promising results, the aim of this study was therefore to evaluate the effect and safety of a combination of curcumin, quercetin, and Vitamin D (NASAFYTOL^®^) in COVID-19 positive hospitalized patients as a supportive treatment to standard-of-care during hospitalization (maximum 14 days).

## 2. Materials and methods

### 2.1. Study design

This study was an exploratory standard-of-care comparative, open-label, parallel two-arm, randomized trial in 50 adult patients who were hospitalized because of a COVID-19 infection. It was a monocentric study carried out at the three sites of the CHIREC hospital group in Belgium between April and October 2021.

### 2.2. Participants

A total of 51 COVID-19 positive hospitalized participants were enrolled in the study, 50 of whom were randomized. Inclusion and exclusion criteria are detailed in the supplementary materials. Oral or written informed consent to participate in the study was obtained from all participants or their authorized legal representatives before enrolment.

Participants had to meet all of the following inclusion criteria: 18 years or older; laboratory-confirmed COVID-19 infection as determined by RT-PCR, or other commercial or public health assay diagnosed not more than 72 h prior to randomization; severity of 3–4–5 according to the WHO 7-point ordinal scale (3: hospitalized, no oxygen therapy; 4: hospitalized, oxygen by mask or nasal prongs; 5: non-invasive ventilation or high-flow oxygen). Participants who met at least one of the following criteria were excluded from the study: any contra-indication to Nasafytol^®^ or its constituents, such as hypersensitivity or allergy to product components; swallowing disorder or inability to take oral capsules; presence of comorbidities that imply a poor prognosis (according to clinical judgment); pregnancy or breastfeeding women; serious or active bacterial infections or documented sepsis by pathogens other than SARS-CoV-2; participation in clinical trials of other products; acute impairment of renal function or nephrolithiasis; liver dysfunction (ALT/AST > 5 times the normal limit), neutropenia (absolute neutrophil count < 500/µl), or thrombocytopenia (platelets < 50,000/µl). Dietary supplements containing one or more ingredients of NASAFYTOL^®^, i.e., Curcuma extract or Vitamin D had to be stopped at study entry.

### 2.3. Randomization

Participants were randomly assigned in a 1:1 ratio to receive Nasafytol^®^ or Vitamin D (Fultium^®^-D3 800; active comparator) in addition to standard-of-care treatment implemented in the hospital according to updated world-wide recommendations (prophylactic enoxaparin, dexamethasone, amoxicillin-clavulanate or ceftriaxone in case of suspicion of bacterial surinfection). Randomization was carried out by blocks of two and four using Castor EDC. No stratification was performed.

### 2.4. Procedures

All patients enrolled in the study were taking either one capsule of Fultium^®^ (EG, Belgium) or eight capsules of Nasafytol^®^ (Tilman, Belgium) per day for 14 days or until they left the hospital or were transferred to the intensive care unit (ICU). Capsules were ingested orally with a glass of water under the supervision of the investigator. The capsule of Fultium^®^ was taken in the morning, while four capsules of Nasafytol^®^ were taken in the morning and four in the evening.

FULTIUM^®^-D3 800 is a blue, softgel capsule that contains 800 UI (20 μg) of vitamin D3 (cholecalciferol). NASAFYTOL^®^ is a green softgel capsule of 1,008 mg, containing a highly bioavailable mixture of turmeric extract (*Curcuma longa* L.) and natural quercetin from *Sophora japonica* L. (pagoda tree or Chinese scholar tree). Each capsule contains 42 mg of curcumin, 65 mg of quercetin, and 2.25 μg of vitamin D3.

Daily follow-up visits were scheduled for 14 days (or < 14 days if discharged earlier). At each visit, COVID-19 WHO ordinal outcome score, temperature, need for oxygen therapy, blood tests (if possible), adverse events (AEs), serious adverse events (SAEs) and death, if any, product intake and counting of capsules for compliance, and concomitant treatments were assessed. At enrolment, medical history and demographic data including birth, gender, ethnicity, weight, height, and BMI were assessed. In addition, at the end of the study, duration of hospitalization was recorded.

### 2.5. Outcomes

Due to the exploratory nature of the study, no primary or secondary outcomes were defined. The following outcome measures were evaluated:

• Change of clinical condition of the patient defined by the COVID-19 WHO ordinal outcome score:
o Time evolution of the COVID-19 WHO ordinal outcome score,
o Score at 7 days and 14 days (or at hospital leave if < 14 days) post randomization,
o The distribution of the COVID-19 WHO ordinal outcome score.
o The absolute and relative changes of the COVID-19 WHO ordinal outcome score compared to baseline at day 7.
• Duration (days) of hospitalization (maximum 14 days).
• Number of transfers to intensive care unit and in-hospital mortality during the trial (maximum 14 days).
• Number of discharged vs. hospitalized participants at day 7 and 14.
• Temperature (fever) in degree Celsius:
o Time to resolution of fever for at least 48 h without antipyretics for 48 h from the first fever period,
o Proportion of patients without fever at day 14 (or at hospital leave if < 14 days).
• Need of oxygen therapy:
o Proportion of patients with need of oxygen therapy at day 14 (or at hospital leave if < 14 days),
o Duration of need of oxygen therapy from the first need of oxygen therapy,
• Tolerance as defined by the number of incidents—AEs and SAEs.
• Compliance using pill count.
• Evolution between baseline and day 14 of blood tests, i.e., C-reactive protein (CRP), hematological values including lymphocyte count: vitamin D serum concentrations.

The investigator was responsible for the detection and documentation of AEs and SAEs throughout the study. The description, date, seriousness, duration, outcome, and relationship of (S)AEs with the study product were collected from the signature of informed consent to the end of study. All AEs and medical history (MH) were coded using the last effective version of MedDRA, and all medications according to ATC/DDD Classifications Index.

In compliance with recommendations of WHO COVID-19 Trial, a Data Safety Monitoring Board (DSMB) composed of two independent experts was responsible for safeguarding the interests of the participants and for enhancing the integrity of the trial. Three DSMB meetings were held during the study (after 25, 50, and 100% of recruitment).

### 2.6. Statistical analysis

Considering a difference between two independent means, two-tails, an alpha of 5%, a difference between two means of 10% and an SD of 10% in each group (effect size of 1) for the endpoint of interest, the total estimated sample size was 46 participants. This has been inflated by 10% to compensate for drop-outs, and a total of 50 participants were recruited for this study. A sample size of 50–100 subjects is also the target size recommended by the WHO for COVID-19 pilot stage studies (23).

Safety analyses and statistical analyses were performed on the full analysis set (FAS) population following the guidelines on statistical principles for clinical trials (ICH E9). There were no differences between the per protocol (PP) population and the FAS population. Statistical analysis results on the PP population would therefore be the same as those derived from the FAS population.

Quantitative variables were summarized using classical descriptive statistics. Mean and standard deviation (± SD) were presented for quantitative variables with a normal distribution. Median and interquartile range (P25–P75) were used for quantitative variables with dissymmetric distribution. Assessment of the normality of quantitative variables distribution was performed numerically by comparing mean and median values and graphically with histograms and quantile-quantile plots. Shapiro–Wilk normality tests completed this investigation of normality. Qualitative variables were assessed using counts and percentage of subjects. Median and interquartile range (P25–P75) were also presented for qualitative ordinal variables.

To detect the presence of potential confounding factors, the homogeneity of demographic data and baseline characteristics was tested between both treatment groups. Chi-squared test was used for qualitative variables while Student’s *t*-test or its non-parametric equivalent was performed for quantitative variables depending on the distribution. Vaccination status as a potential confounding factor was used as adjustment factors using multivariate models.

Statistical significance was achieved at 95% confidence (value of *p* significance < 0.05). Due to exploratory nature of the study, no multiplicity adjustment was implemented. All tests were two-sided and performed on observed data only; missing data were not replaced.

Statistical analyses were performed using SAS Software (version 9.4 for Windows) and using R (version 3.6).

All derived *p*-values were calculated for not adjusted models as well as for multivariate models adjusted by potential confounding factors.

#### 2.6.1. Covid-19 WHO ordinal outcome score

The score at day 7 and 14 (or at hospital leave if < 14 days) was compared between groups using Mann–Whitney’s test. A multivariate linear model was also used to adjust for potential confounding factors.

Time evolution of the COVID-19 WHO ordinal outcome score was summarized using median and interquartile range (P25–P75) in each group at each day and multivariate mixed models with time, groups and interaction between time and groups as covariate were investigated.

#### 2.6.2. Duration of hospitalisation

Duration of hospitalization was analyzed and compared between groups using survival analysis. Not adjusted and adjusted by potential confounding factors Cox survival models were performed to compare duration of hospitalization between groups. In addition, the number of discharged patients at day 7 and at day 14 was compared between groups using a chi-squared test and using a multivariate binary logistic regression adjusted by potential confounding factors.

#### 2.6.3. Transfers to intensive care unit and in-hospital mortality

The number of transfers to intensive care unit and the number of in-hospital deaths was compared between treatments using Fisher’s exact test and using a multivariate binary logistic regression adjusted by potential confounding factors.

#### 2.6.4. Temperature (fever)

The number of patients with no fever at day 14 (or at hospital leave if < 14 days) was compared using a chi-squared test and a multivariate binary logistic regression adjusted by potential confounding factors.

Time to resolution of first fever period was compared between groups using Cox survival models. Resolution of fever was defined as no fever during at least 48 h without antipyretics for 48 h.

#### 2.6.5. Need of oxygen therapy

The need of oxygen therapy at day 14 (or at hospital leave if < 14 days) was compared between groups using a Chi-squared test and a multivariate binary logistic regression adjusted by potential confounding factors.

Time to no need of oxygen therapy from baseline was compared between groups using Cox survival models.

#### 2.6.6. Compliance

Compliance was assessed with the pill count method and was evaluated in percentage of the estimated versus theoretical consumption. Multivariate mixed models with time, groups and interaction between time and groups were performed not adjusted and adjusted for potential confounding factors.

#### 2.6.7. Blood results

Evolution between baseline and the end of the study period (at day 14 or at hospital leave if < 14 days) of routine blood tests, CRP levels and vitamin D concentrations compared between treatments using Mann–Whitney’s tests. Multivariate linear models were performed to adjust for potential confounding factors.

### 2.7. Ethical approval

The study was conducted in accordance with the Declaration of Helsinki and approved by the Ethics Committee of the University Hospital of Brussels (Erasme-ULB) in Belgium (Comité d’Ethique Hospitalo-Facultaire Erasme-ULB; protocol code B4062020000305 on January 15, 2021). The study was registered on clincaltrials.gov with the identifier NCT04844658.

## 3. Results

**Figure 1:**
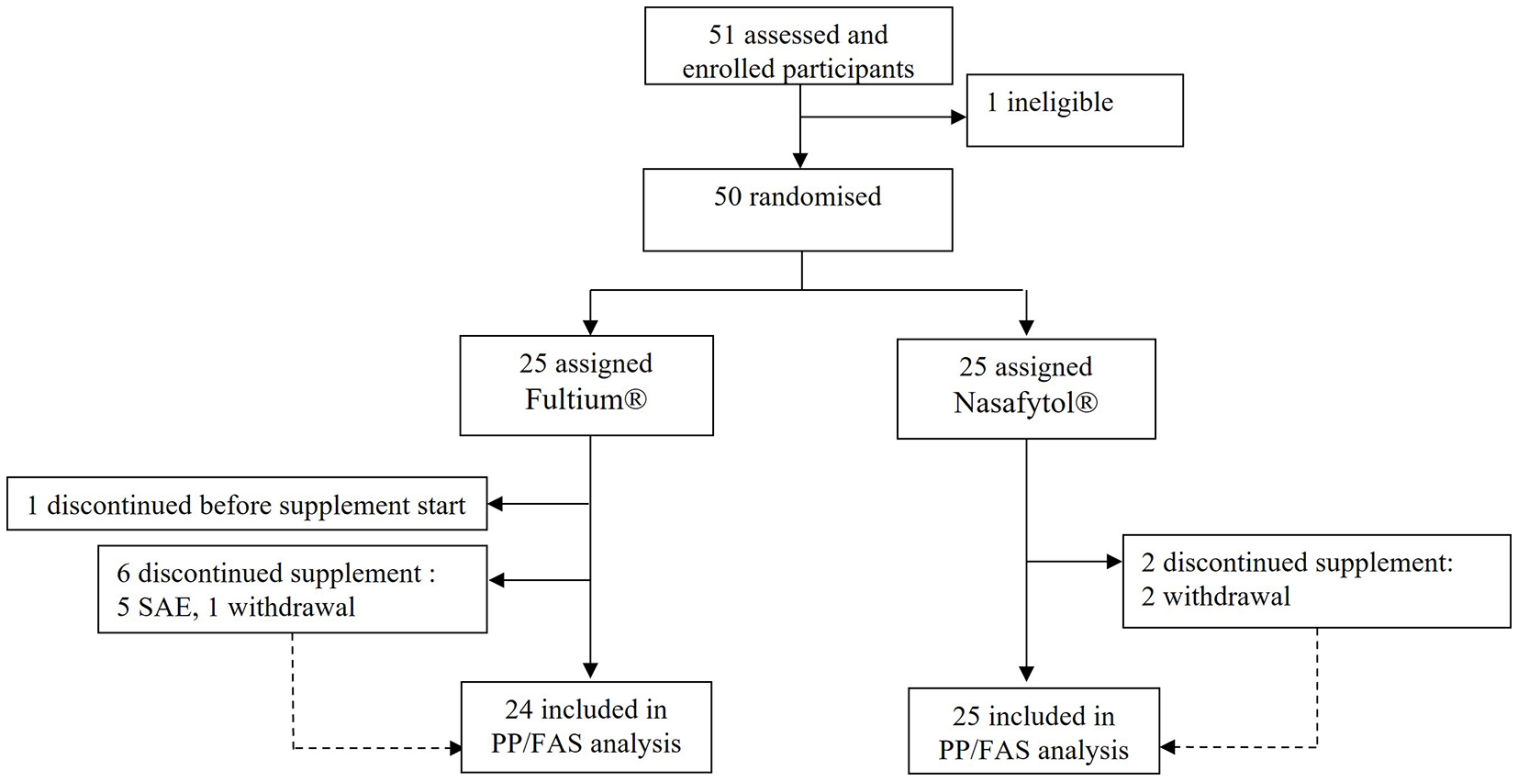
Trial profile.

Fifty-one participants were recruited between April 1^st^ and October 29^th^, 2021. Amongst these, 49 (96.1%) were enrolled in the study without any eligibility violation and took at least one dose of the product, thus constituting the FAS population. All patients included in the FAS population were compliant with the protocol; the PP population was therefore the same as the FAS population (Figure 1). Amongst the 49 participants of the FAS population, 25 (51.0%) received standard treatment and Nasafytol^®^, while 24 (49.0%) participants received standard treatment and Fultium^®^.

Demographic characteristics, including age, sex, height, weight, body-mass index, and ethnic origin were well balanced between the groups (Table 1). The median age was 54 years (range 46–60) in the Nasafytol^®^ group and 58 years (range 52–67) in the Fultium^®^ group. The Nasafytol^®^ group was constituted of 11 (44.0%), the Fultium^®^ group of 15 (62.5%) women. Most participants were of European ethnic origin (18 (72.0%) in Nasafytol^®^ and 20 (83.3%) in Fultium^®^ group). Interestingly, the median BMI was 29.1 (26.9–32.8) kg/m^2^ in the Nasafytol^®^ group, and 29.7 (25.4–33.0) kg/m^2^ in the Fultium^®^ group, indicating that all participants were overweight or obese.

**Table 1:** Descriptive statistics of the demographic characteristics globally and by group—FAS/PP population—*N* = 49.

				Treatment				
Variable	Categories	All		Fultium^®^		Nasafytol^®^		
		*N*	Number (%)	*N*	Number (%)	*N*	Number (%)	*p*-Value
Age (year)	Median (P25-P75)	49	55.0 (50.0–64.0)	24	58.0 (52.0–67.0)	25	54.0 (46.0–60.0)	0.15
Sex		49		24		25		0.19
	Female		26 (53.1)		15 (62.5)		11 (44.0)	
	Male		23 (46.9)		9 (37.5)		14 (56.0)	
Height (cm)	Median (P25-P75)	49	168.0 (162.0–176.0)	24	167.0 (159.5–173.5)	25	172.0 (162.0–177.0)	0.37
Weight (kg)	Median (P25-P75)	49	84.0 (72.8–95.0)	24	85.0 (70.5–96.0)	25	82.0 (73.5–93.2)	0.98
BMI (kg/m^2^)	Median (P25–P75)	49	29.2 (26.2–32.8)	24	29.7 (25.4–33.0)	25	29.1 (26.9–32.8)	0.98
Ethnic		49		24		25		0.39
	African		6 (12.2)		3 (12.5)		3 (12.0)	
	European		38 (77.6)		20 (83.3)		18 (72.0)	
	Other		5 (10.2)		1 (4.2)		4 (16.0)	

The median oxygen saturation at baseline was 94.0 (94.0–96.0) % for participants in the Nasafytol^®^ group and 94.0 (92.0–96.0) % for participants included in the Fultium^®^ group. The median oxygen flow rate was 3.5 (2.0–5.5) L/min for participants receiving Nasafytol^®^ and 2.5 (2.0–6.0) L/min for participants treated with Fultium^®^. There was no significant difference between groups for either parameter (Supplementary Table 1). While temperature and the number of concomitant treatments per patient was different between the two groups, the median number of medical histories per patient was comparable in both groups. The proportion of patients with at least one vaccination dose was however significantly higher in the Fultium^®^ group than in Nasafytol^®^ group (37.5 vs. 8.0%, *p* = 0.013; Supplementary Table 1). For this reason, the *p*-values of further analyses were adjusted for the vaccine status.

**Figure 2:**
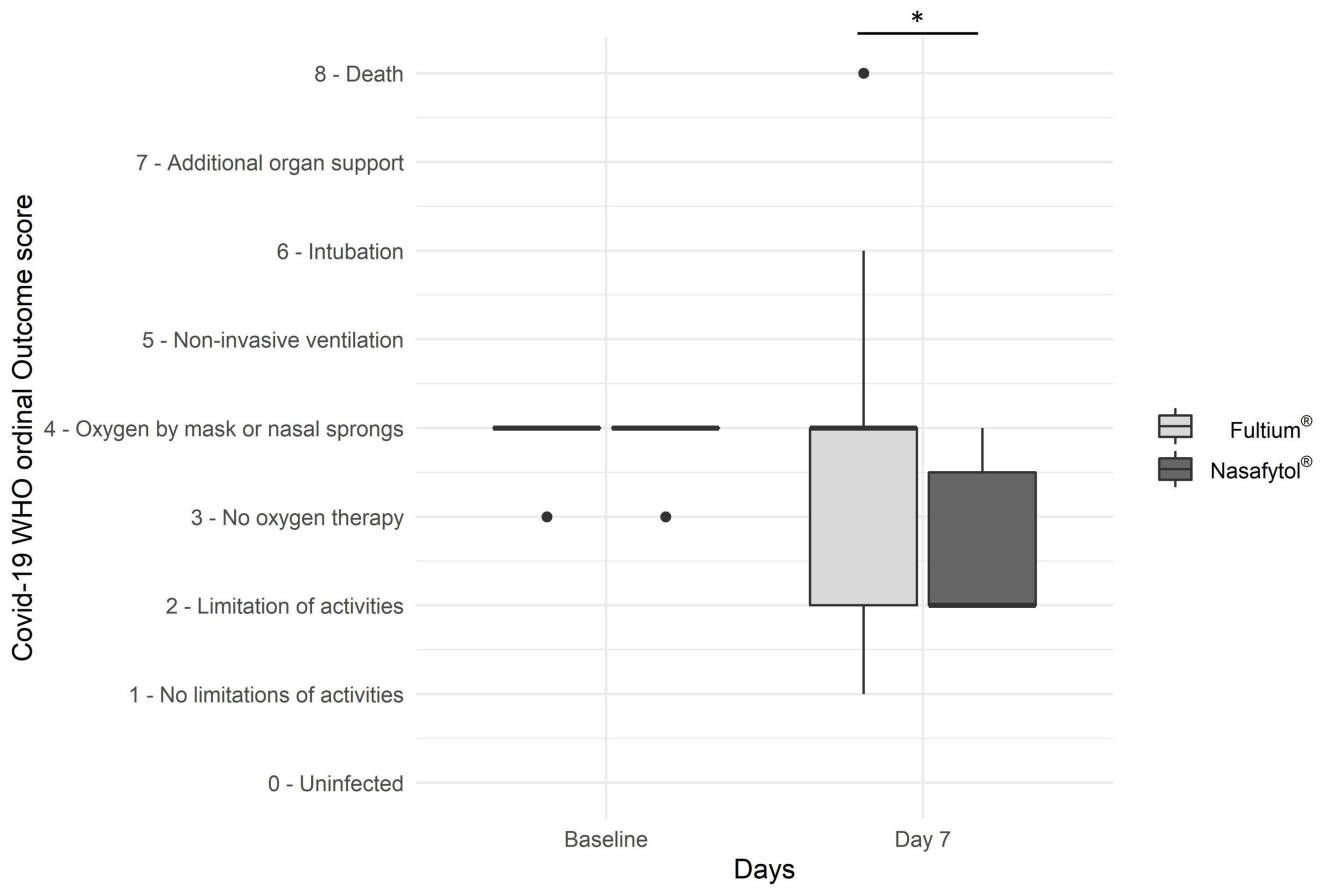
Efficacy outcomes of Nasafytol^®^ versus Fultium^®^ at day 7: Boxplot of the COVID-19 WHO ordinal outcome score. Adjusted value of *p*: ^*^p < 0.05.

Interestingly, the median COVID-19 WHO ordinal outcome score was significantly lower in the Nasafytol^®^ group than in the Fultium^®^ group 2.0 (2.0–4.0) vs. 4.0 (2.0–4.0) at day 7 (Supplementary Table 2; Figure 2). Moreover, the absolute and the relative change of the COVID-19 WHO ordinal outcome score at day 7 compared to baseline was significantly different between the two groups. In the Nasafytol^®^ group, patients presented a median absolute improvement of 2.0 (0.0–2.0) and a relative improvement of 50.0 (0.0–50.0) % compared to baseline while in the Fultium^®^ group, the absolute change was equal to 0.0 (−0.5–2.0) and the relative change was equal to 0.0 (−16.7–50.0) % (Table 2). At day 14 (or at hospital leave if < 14 days), no significant differences were observed for the median COVID-19 WHO ordinal outcome score for patients still hospitalized; neither the absolute nor the relative change of COVID-19 WHO ordinal outcome score compared to baseline were significantly different between the two groups. As only 4 participants (two in each group) remained at day 14, no longitudinal analysis could be performed. Similarly, no significant difference was observed between the two groups regarding the number of patients who had a fever or required oxygen therapy at day 14 (or at hospital leave if < 14 days), or the time to resolution of the first fever period or to no need of oxygen therapy. No significant difference in the evolution of blood results, including CRP levels, was observed between the two groups.

**Table 2:** Descriptive statistics of the absolute change and relative change of the COVID-19 WHO ordinal outcomes score at day 7 from baseline globally and by treatment—FAS/PP population—*N* = 46.

			Treatment					
			Fultium^®^		Nasafytol^®^			
Change at day 7	*N*	Median (P25-P75)	*N*	Median (P25-P75)	*N*	Median (P25-P75)	*p*-Value	Adjusted *p*-value^*^
Absolute	46	1.5 (0.0–2.0)	24	0.0 (−0.5–2.0)	22	2.0 (0.0–2.0)	0.02	0.02
Relative (%)	46	41.7 (0.0–50.0)	24	0.0 (−16.7–50.0)	22	50.0 (0.0–50.0)	0.02	0.02

* Adjusted for vaccine status.

**Figure 3:**
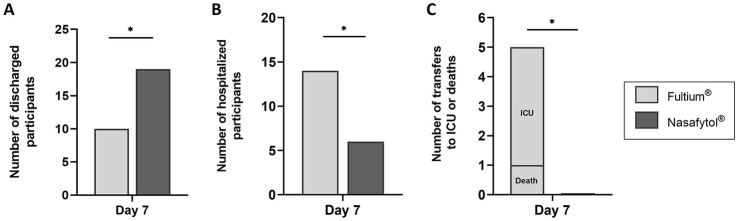
Efficacy outcomes of Nasafytol^®^ versus Fultium^®^ at day 7. **(A)** Number of discharged participants. **(B)** Number of hospitalized participants. **(C)** Number of transfers to the ICU or deaths. Adjusted value of *p*: ^*^p < 0.05.

In addition, the number of discharged participants was significantly higher in the Nasafytol^®^ group than in the Fultium^®^ group (19 vs. 10) at day 7. Accordingly, the number of hospitalized participants was significantly lower in the Nasafytol^®^ group than in the Fultium^®^ group (6 vs. 14; Figure 3A,B; Supplementary Table 3). Moreover, the number of participants who died or were transferred to the ICU by day 7 was significantly higher in the Fultium^®^ group than in the Nasafytol^®^ group (4 transfers to ICU and 1 death vs. 0 transfers to ICU and 0 deaths; Figure 3C; Supplementary Table 3). At the end of the study, 23 participants had been discharged in the Nasafytol^®^ group, and 17 in the Fultium^®^ group. This difference was however not statistically different, identical to the median duration of hospitalization (Supplementary Table 4).

Compliance was 100% except for the first day when some patients received Nasafytol^®^ in the evening instead of in the morning.

Thirty-eight AEs were recorded during the study; 22 (57.9%) AEs occurred in Fultium^®^ group and 16 (42.1%) AEs occurred in Nasafytol^®^ group. Among these, five SAEs were recorded during the study, all in Fultium^®^ group. As shown in Supplementary Table 5, acute respiratory failure was the most frequently observed SAE [4 (80.0%)] while COVID-19 respiratory infection was observed once (20.0%). While the distribution of the number of AEs per participant was comparable between groups, the proportion of participants with SAEs was significantly higher in the Fultium^®^ group (5 SAEs) than in the Nasafytol^®^ group (0 SAEs; Table 3).

**Table 3:** Distribution of the number of AES and SAEs per patient globally and by group—safety population—*N* = 49.

				Treatment groups				
		All		Fultium^®^		Nasafytol^®^		
Variable	Categories	*N*	Number (%)	*N*	Number (%)	*N*	Number (%)	Not adjusted *p*-value
Number of AEs per patient		49		24		25		0.60
	0		25 (51.0)		13 (54.2)		12 (48.0)	
	1		17 (34.7)		7 (29.2)		10 (40.0)	
	2		5 (10.2)		2 (8.3)		3 (12.0)	
	3		1 (2.0)		1 (4.2)		0 (0.0)	
	8		1 (2.0)		1 (4.2)		0 (0.0)	
Number of SAEs per patient		49		24		25		0.023
	0		44 (89.8)		19 (79.2)		25 (100.0)	
	1		5 (10.2)		5 (20.8)		0 (0.0)	

## 4. Discussion

This is, to the best of our knowledge, the first randomized, controlled trial to test the effect and safety of a combination of vitamin D3, curcumin and quercetin (Nasafytol^®^) as supplement to standard-of-care treatment for COVID-19 in hospitalized adult patients. We found a significant risk reduction for serious outcomes as being evidenced by a reduced number of deaths or transfers to the ICU. In addition, the clinical condition had improved as evidenced by a reduced COVID-19 WHO ordinal outcome score, and the number of discharged participants after 7 days of follow-up had increased.

Our results are consistent with a recent pilot open-label randomized trial carried out in Pakistan. The authors suggest a possible therapeutic effect of an oral combination of vitamin D3, curcumin and quercetin when taken as a supplement to paracetamol and/or antibiotics in ambulatory patients with early-stage mild to moderate COVID-19 symptoms (22). They observed an accelerated clearance of SARS-CoV-2 viral particles, CRP levels as well as accelerated, albeit not statistically significant, symptoms resolution compared to participants who did not receive any supplement in addition to standard-of-care treatment (22).

As vitamin D3 deficiency seems to be an important predictor for severe outcomes of SARS-CoV-2 infections, and associations of low serum vitamin D3 levels with severity of the disease have been reported (24), supplementation of COVID-19 patients with vitamin D3 has been widely discussed. Further, its potential role in decreasing the risk of a COVID-19 infection has been described by a recent review (10). While an improvement of acute symptoms has been observed in patients with early-stage mild to moderate COVID-19 disease after adjuvant supplementation with vitamin D3 (25), supplementation with a single high dose of vitamin D3 was not able to significantly reduce the length of hospitalization in patients with severe COVID-19 (11). Its combination with other supplements such as curcumin and quercetin therefore seemed an interesting approach. Since the beginning of this trial in April 2020, a positive effect of adjuvant supplementation with curcumin, including faster recovery from mild to severe symptoms, shorter hospitalization, or fewer deaths, has been observed in several randomized clinical trials (26). Similarly, beneficial effects have also been described after adjuvant supplementation with quercetin. Several randomized clinical trials reported accelerated recovery of acute symptoms, shorter hospitalization, less transfers to the ICU, and a reduced number of deaths (27). The originality of our approach was to combine these three natural compounds and to introduce them in addition to standard of care in treatment of hospitalized COVID-19 patients.

The underlying mechanisms of vitamin D3, curcumin and quercetin for COVID-19 disease remain uncertain. Vitamin D, *via* its active metabolite 1,25(OH)2D, regulates adaptive immunity by limiting the maturation of dendritic cells, limiting their ability to present antigen to T cells, and inhibiting proinflammatory processes (28). Curcumin is known to inhibit pro-inflammatory cytokines such as Il-1, IL-2, IL-6, IL-8, IL-10, IL-11, IL-12, IL-17, tumor necrosis factor alpha (TNF-α), and interferon-gamma (IFN-γ), as well as chemokines including nuclear factor kappa-light-chain enhancer of activated B cells (NFκB), monocyte chemoattractant protein 1 (MCP-1), macrophage inflammatory protein 1α (MIP-1α), cyclooxygenase (COX), plasminogen activator inhibitor-1 (PAI-1), and caspase-3 (18). Moreover, it has recently been shown to play a role in the SARS-CoV-2-induced cytokine storm inhibition through deactivation of the mitogen-activated protein kinases (MAPK)/NFκB signaling pathways in epithelial cells *in vitro* (29). Quercetin also inhibits pro-inflammatory cytokines such as IL-1β, IL-6, IFN-γ, and TNF-α (30). In combination with dasatinib it has recently been shown to reduce inflammation in SARS-CoV-2 infected hamsters and mice (31). Additional *in vitro* and *in vivo* studies are needed to dissect the mechanisms by which vitamin D3, curcumin and quercetin contribute to an amelioration of COVID-19 symptoms and exert a beneficial therapeutic effect. Despite these anti-inflammatory properties, our study failed to demonstrate an effect of Nasafytol^®^ on the inflammatory blood markers. This was in disagreement with the observation of the recent Khan et al. (22) study which showed a significant decrease of serum CRP levels after adjunction of Vitamin D/quercetin/curcumin combination. It would also be interesting to investigate the combinatorial effect of vitamin D3, curcumin and quercetin on overweight and obese subject who are especially at risk of a severe outcome when suffering from COVID-19 (32).

Another key result of our study is the absence of adverse effect observed with Nasafytol^®^. The non-toxicity and safety of curcumin for chronic and acute use is very well documented. In addition to its traditional use in food, clinical trials support its safety for doses up to 12 g per day (20). Similarly, quercetin is considered a food ingredient, with a mean daily intake of 16–50 mg in occidental countries. Its safety for a supplementation with up to 1 g per day for 3 months has been shown in clinical trials (21). Finally, daily supplementation with vitamin D3 is safe with a recommended dietary allowance (RDA) of 600 IU (33). As for the combination of the three, a test for acute oral toxicity of Nasafytol^®^ in rats according to OECD guidelines for the testing of chemicals (test n°423: Acute Oral Toxicity—Acute Toxic Class Method; 17/12/2001) did not reveal any mortality, did not influence the weight curves, and did not cause any macroscopically visible organic or tissue alterations. Moreover, the nutrivigilance of Nasafytol^®^ did not reveal any side effects in 9 years of use on different markets in Belgium (750,000 capsules per year), Morocco (1 million capsules per year), Finland (200,000 capsules per year), Poland (200,000 capsules per year) and Latvia (100,000 capsules per year). Given vitamin D3’s, curcumin’s and quercetin’s excellent safety and tolerability profile, in addition to their beneficial effects observed in the present study and in the literature, their combination provides a highly interesting alternative for the supplementary treatment of mildly to moderately, and even severely affected COVID-19 patients. Considering the immunosuppressive side effects of other anti-inflammatory or immunomodulatory treatments currently used for COVID-19, such as corticosteroids, tocilizumab or bariticinib, this is of particular interest.

Limitations of our trial are related to the small number of participants, and the fact that it was not a double-blinded trial. The major limitation is however that the design of the study did not permit to detect a significant effect at day 14 as most participants had left the study by that time, either because they had been discharged, transferred to the ICU or even died. This was partly due to the fact that COVID-19 was still not well characterized at the time of the study, thus making the estimation of the correct duration of follow-up more difficult. Our understanding of COVID-19 disease progression and outcomes has evolved since the beginning of this trial in April 2020. The promising results of the present study provide, however useful information for the design of a larger double-blind randomized controlled clinical trial, particularly regarding the choice of the primary outcome and the duration of follow-up. In addition, the evaluation of biomarkers as part of any future clinical trial would be helpful to clarify the underlying mechanisms of action of a combination of these supplements in the context of COVID-19 disease.

Based on these findings, we can conclude that Nasafytol^®^ used as supplement to standard of care management of COVID-19 hospitalized patients is a safe approach that allows to reduce the hospitalization time for COVID-19 infection, improves the clinical status of patients suffering from a moderate-to-severe form of the disease.

## Data availability statement

The original contributions presented in the study are included in the article/Supplementary material, further inquiries can be directed to the corresponding author.

## Ethics statement

The studies involving human participants were reviewed and approved by Ethics Committee of the University Hospital of Brussels (Erasme-ULB) in Belgium (Comité d’Ethique Hospitalo-Facultaire Erasme-ULB) (protocol code B4062020000305 on January 15, 2021). The patients/participants provided their written informed consent to participate in this study.

## Author contributions

BC and YH: conceptualization. JG, BC, JH, and YH: methodology. A-FD and JM: statistical analysis. JG: investigation. MU and YH: writing—original draft preparation. JG, BC, JH, SP, A-FD, and JM: writing—review and editing. MU, BC, and JH: project administration. JG, MU, BC, JH, SP, A-FD, JM, and YH had full access to all the data in the study and had final responsibility for the decision to submit for publication. BC, JH, and YH accessed and verified the data. All authors contributed to the article and approved the submitted version.

## Funding

The study was funded by Tilman SA and performed by an independent CRO according to ICH guidelines and the Declaration of Helsinki. The funder of the study had no role in study design, data collection, data analysis, data interpretation, or writing of the report.

## Conflict of interest

YH is the founder and president of Artialis SA, a spin-off company of the University of Liège. YH has also received consulting and speaker fees from Tilman SA, Nestlé, Laboratoire Expanscience, Heel, Megalab, Genequine, LABHRA, and Biose. MU, BC, JH, and SP are employees of Artialis SA.

The remaining authors declare that the research was conducted in the absence of any commercial or financial relationships that could be construed as a potential conflict of interest.

## Publisher’s note

All claims expressed in this article are solely those of the authors and do not necessarily represent those of their affiliated organizations, or those of the publisher, the editors and the reviewers. Any product that may be evaluated in this article, or claim that may be made by its manufacturer, is not guaranteed or endorsed by the publisher.
