# Supplementary material for: NASAFYTOL® supplementation in adults hospitalized with COVID-19 infection: results from an exploratory open-label randomized controlled trial
Source: Front Nutr. 2023 Jun 22;10:1137407. doi: 10.3389/fnut.2023.1137407 (PMC10324407; doi:10.3389/fnut.2023.1137407)
Supplement: Supplementary file 1 [file Table_1.DOCX]

Supplementary Material

**NASAFYTOL® in adults hospitalized with COVID-19 infection: results from an exploratory open-label randomized controlled trial**

**Jean Gérain^1^, Melanie Uebelhoer^2^*, Bérénice Costes^2^, Julie Herman^2^, Sandra Pietri^2^, Anne-Françoise Donneau^3^, Justine Monseur^3^, Yves Henrotin^2^**

*** Correspondence:** Melanie Uebelhoer: [melanie.uebelhoer@artialis.com](mailto:melanie.uebelhoer@artialis.com)

# Supplementary Tables

| Supplementary table 1: Descriptive statistics of baseline conditions globally and by treatment – FAS/PP population – N=49 | | | | | | | | |
| --- | --- | --- | --- | --- | --- | --- | --- | --- |
|  | |  | | Treatment | | | |  |
|  | | All | | Fultium® | | Nasafytol® | |  |
| Variable | Categories | N | Number (%) | N | Number (%) | N | Number (%) | P-value |
| Vaccine status* |  | 49 |  | 24 |  | 25 |  | *0*.*013* |
|  | No |  | 38 (77.6) |  | 15 (62.5) |  | 23 (92.0) |  |
|  | Yes |  | 11 (22.4) |  | 9 (37.5) |  | 2 (8.0) |  |
| Oxygen saturation (%) | Median (P25-P75) | 49 | 94.0 (93.0-96.0) | 24 | 94.0 (92.0-96.0) | 25 | 94.0 (94.0-96.0) | 0.27 |
| Oxygen flow rate (L/min) | Median (P25-P75) | 49 | 3.0 (2.0-6.0) | 24 | 2.5 (2.0-6.0) | 25 | 3.5 (2.0-5.5) | 0.36 |
| Medical histories | Median (P25-P75) | 49 | 2.0 (1.0-3.0) | 24 | 2.0 (1.0-3.0) | 25 | 2.0 (1.0-3.0) | 0.71 |
| Concomitant medications | Median (P25-P75) | 49 | 5.0 (3.0-7.0) | 24 | 6.0 (3.5-9.5) | 25 | 4.0 (2.0-5.0) | *0*.*021* |
| Temperature (fever) | No | 49 | 23 (46.9) | 24 | 10 (41.7) | 25 | 19 (76.0) | *0*.*015* |
|  | Yes |  | 26 (53.1) |  | 14 (58.3) |  | 6 (24.0) |  |
| * At least one dose | | | | | | | | |

| Supplementary table 2: Distribution of the COVID-19 WHO ordinal outcome score at day 7 globally and by treatment – FAS/PP population – N=46 (drop-out patients were not considered). | | | | | | | | |
| --- | --- | --- | --- | --- | --- | --- | --- | --- |
|  |  | | Treatment | | | |  |  |
|  | All | | Fultium® | | Nasafytol® | |  |  |
| Score at day 7 | N | Number (%) | N | Number (%) | N | Number (%) | P-value | Adjusted P-value* |
| Median (P25-P75) | 46 | 2.0 (2.0-4.0) | 24 | 4.0 (2.0-4.0) | 22 | 2.0 (2.0-4.0) | 0.04 | 0.03 |
| 0-Uninfected |  | 0 (0.0) |  | 0 (0.0) |  | 0 (0.0) |  |  |
| 1-No limitation of activities |  | 1 (2.2) |  | 1 (4.2) |  | 0 (0.0) |  |  |
| 2-Limitation of activities |  | 25 (54.3) |  | 9 (37.5) |  | 16 (72.7) |  |  |
| 3-No oxygen therapy |  | 1 (2.2) |  | 1 (4.2) |  | 0 (0.0) |  |  |
| 4-Oxygen by mask or nasal prongs |  | 14 (30.4) |  | 8 (33.3) |  | 6 (27.3) |  |  |
| 5-Non-invasive ventilation |  | 0 (0.0) |  | 0 (0.0) |  | 0 (0.0) |  |  |
| 6-Intubation |  | 4 (8.7) |  | 4 (16.7) |  | 0 (0.0) |  |  |
| 7-Additional organ support |  | 0 (0.0) |  | 0 (0.0) |  | 0 (0.0) |  |  |
| 8-Death |  | 1 (2.2) |  | 1 (4.2) |  | 0 (0.0) |  |  |

* adjusted for vaccine status

| Supplementary table 3: Distribution of the number of discharged, hospitalized, and transfers to ICU or death at day 7 – FAS/PP population – N=49 | | | | | | | | | |
| --- | --- | --- | --- | --- | --- | --- | --- | --- | --- |
|  | |  | | Treatment | | | |  |  |
|  | | All | | Fultium® | | Nasafytol® | |  |  |
| Status at day 7 | Categories | N | Number (%) | N | Number (%) | N | Number (%) | P-value | Adjusted  P-value* |
| Discharged |  | 49 |  | 24 |  | 25 |  | 0.02 | 0.03 |
|  | No (Follow-up or SAE) |  | 20 (40.8) |  | 14 (58.3) |  | 6 (24.0) |  |  |
|  | Yes |  | 29 (59.2) |  | 10 (41.7) |  | 19 (76.0) |  |  |
| Hospitalized |  | 49 |  | 24 |  | 25 |  | 0.02 | 0.03 |
|  | No |  | 29 (59.2) |  | 10 (41.7) |  | 19 (76.0) |  |  |
|  | Yes (Follow-up or SAE) |  | 20 (40.8) |  | 14 (58.3) |  | 6 (24.0) |  |  |
| Transfer to ICU or death |  | 49 |  | 24 |  | 25 |  | 0.02 | - |
|  | No  Yes |  | 44 (89.8)  5 (10.2) |  | 19 (79.2)  5 (20.8) |  | 25 (100.0)  0 (0.0) |  |  |

* adjusted for vaccine status

| Supplementary table 4: Distribution and comparisons between groups of number of discharged patients during the study period – FAS/PP population – N=49 | | | | | | | | | |
| --- | --- | --- | --- | --- | --- | --- | --- | --- | --- |
|  |  |  | | Treatment groups | | | |  |  |
|  |  | All | | Fultium® | | Nasafytol® | |  |  |
| Variable | Categories | N | Number (%) | N | Number (%) | N | Number (%) | Not adjusted  P-value | Adjusted  P-value* |
| Discharged at day 14 |  | 49 |  | 24 |  | 25 |  | 0.06 | 0.10 |
|  | No |  | 9 (18.4) |  | 7 (29.2) |  | 2 (8.0) |  |  |
|  | Yes |  | 40 (81.6) |  | 17 (70.8) |  | 23 (92.0) |  |  |
| Variable | Percentile | N | Point estimate  (95%CI) | N | Point estimate  (95%CI) | N | Point estimate  (95%CI) | Not adjusted p-value | Adjusted p-value |
| Duration (days) |  | 49 |  | 24 |  | 25 |  | 0.44 | 0.48 |
|  | P25 |  | 3.0 (2.0-4.0) |  | 3.0 (1.0-5.0) |  | 3.0 (1.0-4.0) |  |  |
|  | Median |  | 5.0 (4.0-7.0) |  | 8.0 (3.0-8.0) |  | 5.0 (3.0-6.0) |  |  |
|  | P75 |  | 8.0 (6.0-10.0) |  | 9.0 (8.0-.) |  | 7.0 (5.0-10.0) |  |  |

* adjusted for vaccine status

| Supplementary table 5: Listing of AEs and SAEs by treatment and by decreasing order of frequency – Safety population – N=38 adverse events | | |
| --- | --- | --- |
| Treatment groups | Adverse Event | N (%) |
| Fultium® (N=22 adverse events) | Acute Respiratory Failure | 4 (18.2) |
|  | Diarrhoea Infectious | 3 (13.6) |
|  | Headache | 2 (9.1) |
|  | Localised Pruritus | 2 (9.1) |
|  | Coughing | 1 (4.5) |
|  | COVID-19 Respiratory Infection | 1 (4.5) |
|  | CRP Positive | 1 (4.5) |
|  | Function Liver Abnormal | 1 (4.5) |
|  | Function Pulmonary Decreased | 1 (4.5) |
|  | Hyperglycaemia | 1 (4.5) |
|  | LDH Increased | 1 (4.5) |
|  | Stomach Burning Sensation Of | 1 (4.5) |
|  | Stress | 1 (4.5) |
|  | Tachycardia | 1 (4.5) |
|  | Vomiting | 1 (4.5) |
| Nasafytol® (N=16 adverse events) | Feeling Anxious | 2 (12.5) |
|  | Fever | 2 (12.5) |
|  | Arterial Hypertension | 1 (6.3) |
|  | Bacterial Infection | 1 (6.3) |
|  | Cloudy Urine | 1 (6.3) |
|  | Constipation | 1 (6.3) |
|  | Fibrin D Dimer Increased | 1 (6.3) |
|  | Gastritis | 1 (6.3) |
|  | Headache | 1 (6.3) |
|  | Hypokalaemia | 1 (6.3) |
|  | Obstipation | 1 (6.3) |
|  | Pharyngitis Streptococcal | 1 (6.3) |
|  | Transient Nocturnal Oxygen Desaturation | 1 (6.3) |
|  | Vertigo | 1 (6.3) |
| Treatment groups | Serious Adverse Event | N (%) |
| Fultium® (N=5 serious adverse events) | Acute Respiratory Failure | 4 (80.0) |
|  | COVID-19 Respiratory Infection | 1 (20.0) |
